# Pathogenesis from the microbial-gut-brain axis in white matter injury in preterm infants: A review

**DOI:** 10.3389/fnint.2023.1051689

**Published:** 2023-03-16

**Authors:** Yuqian Wang, Jing Zhu, Ning Zou, Li Zhang, Yingjie Wang, Mengmeng Zhang, Chan Wang, Liu Yang

**Affiliations:** Department of Pediatrics, The Second Hospital of Dalian Medical University, Dalian, Liaoning, China

**Keywords:** premature infants, brain damage, microbial-gut-brain axis, white matter injury, neurological, neurology

## Abstract

White matter injury (WMI) in premature infants is a unique form of brain injury and a common cause of chronic nervous system conditions such as cerebral palsy and neurobehavioral disorders. Very preterm infants who survive are at high risk of WMI. With developing research regarding the pathogenesis of premature WMI, the role of gut microbiota has attracted increasing attention in this field. As premature infants are a special group, early microbial colonization of the microbiome can affect brain development, and microbiome optimization can improve outcomes regarding nervous system development. As an important communication medium between the gut and the nervous system, intestinal microbes form a microbial-gut-brain axis. This axis affects the occurrence of WMI in premature infants *via* the metabolites produced by intestinal microorganisms, while also regulating cytokines and mediating oxidative stress. At the same time, deficiencies in the microbiota and their metabolites may exacerbate WMI in premature infants. This confers promise for probiotics and prebiotics as treatments for improving neurodevelopmental outcomes. Therefore, this review attempted to elucidate the potential mechanisms behind the communication of gut bacteria and the immature brain through the gut-brain axis, so as to provide a reference for further prevention and treatment of premature WMI.

## Introduction

With the gradual improvement in neonatal treatment, the survival rate of small- gestational-age preterm infants has improved greatly, but the long-term neurological prognosis is still not optimal; many surviving infants, especially those who are very premature, develop long-term neurological dysfunction associated with social and economic burden (Raju et al., 2017; Duncan and Matthews, 2018). For example, reports suggest that approximately 10% of preterm infants have varying degrees of cerebral palsy, with symptoms ranging from mild to severe spastic dyskinesia (Beaino et al., 2010). In addition, 25–50% of preterm infants experience extensive cognitive impairment, audiovisual impairment, social behavior disorders, and attention, and learning disabilities (Glass et al., 2008; Anderson et al., 2011). White matter injury (WMI) in preterm infants is a unique and common form of brain injury, especially in those born before 28 weeks of gestation (Rantakari et al., 2021). As changes in the intestinal microbial structure of preterm infants may affect the development and function of the brain, the risk of WMI in premature infants may be reduced by accelerating the colonization of intestinal microbes. Moreover, it is known that metabolites produced by intestinal microorganisms can regulate the immune inflammatory response characterized by the activation of microglia, and the deficiency of these metabolites may exacerbate WMI in premature infants. Intestinal microorganisms can also regulate damage caused by oxidative stress (OS), which ultimately affects the differentiation of precursor oligodendrocytes (OLs) and myelin sheath formation leading to white matter damage in premature infants. Deficiencies of microbiota and microbial metabolites may exacerbate WMI in premature infants. Here, we review the interactions between WMI and gut microbes and elucidate the underlying mechanisms by which gut bacteria and immature brains communicate with each other. Currently, the treatment of WMI is limited to preventive management rather than targeted therapy. We review the latest evidence on microbiological interventions and treatments for WMI in preterm infants, such as prebiotics and probiotics, and attempt to analyze the neuroprotective effects of prebiotics and probiotics on very preterm infants.

## Brain development and changes in gut microbiome

Neurodevelopment is a complicated and fluctuant process controlled by many factors. Brain development begins as early as the embryonic stage. During this period, the external environment influences fetal neural development through maternal immunity and metabolism. Factors that break maternal homeostasis such as infection, malnutrition, and prenatal stress are associated with several neurodevelopmental diseases, and any imbalance in maternal intestinal microecology may be a bridge between the two. Neurodevelopment continues during the neonatal period and includes morphological changes, cell differentiation, and function acquisition (Borre et al., 2014). Studies have shown that the gut microbiome is involved in the processes of perinatal brain development including neurogenesis, microglia maturation, blood-brain barrier development, and myelination (Ogbonnaya et al., 2015; Hoban et al., 2016; Erny et al., 2017; Farzi et al., 2018). One study showed that germ-free (GF) mice showed similar behavior and gene expression when exposed to gut microbes in early life, but did not change behavior when the gut microbes were reconstructed in adult GF mice, suggesting that there may be an optimal window of time for the treatment of neurodysplasia *via* the regulation of gut microecology (Borre et al., 2014). Maturation of gut microbes also occurs early in life. The period of change of intestinal microbes coincides with the period of rapid brain development in infants, and the maturation of intestinal microbes parallels neural development. The first 3 years of life represents a particularly important developmental period for the central nervous system (CNS), with the period of extensive synaptic formation and myelination coinciding with that of gut flora maturation.

The number of microbial communities that colonize the intestine is as high as 10^13^–10^14^, and the total amount of genetic data they contain is 100 times that of humans (Kurokawa et al., 2007; Rutayisire et al., 2016). Overall, the microbiome plays a role in regulating homeostasis. Most of these communities colonize the colon, and a small portion colonizes the small intestine and stomach (Sender et al., 2016). However, there is no consensus on whether fetuses contain bacteria. Regardless, early life exposure, specifically that received during pregnancy and lactation, leads to changes in the offspring’s microbiome throughout their life (Stout et al., 2013). An increasing number of studies have shown that the fetus may acquire initial bacterial colonization *in utero* through the placenta or *via* swallowing amniotic fluid, subsequently forming complex intestinal flora after birth (Ortega et al., 2016). The initial colonization of the intestinal microbiome is typically represented by beneficial and non-pathogenic symbiotic bacteria transmitted by the mother (Bokulich et al., 2016; Yassour et al., 2016) such as *Bifidobacterium*, *Clostridium*, *Lactobacillus*, *Bacteroides*, and others. As the newborn matures, the bacteria proliferate rapidly, consuming a large amount of oxygen in the intestine and forming an environment conducive to the colonization of anaerobic bacteria. Overall, it takes 2–3 years for the intestinal tract to stabilize in terms of type and number of bacteria (Chu et al., 2017). During the first year of life, infants experience weaning, conversion to solid food intake, and rapid brain development. The anaerobic bacteria then take control of the gut, and by 3 years of age, the diversity of *Bacteroides* and *Firmicutes* is increased to numbers similar to those found in adults.

From the 20th week of gestation, the cerebral cortex and gray matter volume begin to increase, neurogenesis takes place, axons and dendrites develop, synapses begin to form, and myelin is formed, while premature infants lose this critical period of normal brain development and maturity *in utero*. Despite many neonates in neonatal intensive care units being given probiotics daily, the establishment of a mutually beneficial relationship between the gut microbiome and its host in very premature infants appears to be impaired (Alcon-Giner et al., 2020). Most premature infants are transferred to a neonatal intensive care unit for treatment after birth, disrupting the normal colonization of the microbiome during its critical period. Risk factors for this disruption include premature rupture of membranes, maternal infection, cesarean section, prophylactic use of antibiotics, inability to breastfeed, mechanical ventilation, delayed enteral nutrition, and prolonged fasting. Due to excessive confounding factors, the influence of delivery and feeding modes of premature infants on intestinal flora requires further study. However, the influence of gestational age on intestinal flora is confirmed at present (Cryan et al., 2019). For example, the results of a Norwegian study that collected fecal samples from 519 infants (including 160 preterm infants) at 4, 10, and 12 months for intestinal microbiota sequencing and metabolite analysis showed that preterm infants have a unique intestinal microflora in the early postnatal period (Dahl et al., 2018). Moreover, preterm infants have a lower degree of bacterial diversity than healthy full-term newborns, with *Enterococcus*, *Escherichia coli*, *Staphylococcus*, *Streptococcus*, and *Clostridium* being the most prevalent, with colonization of beneficial strains such as *Bifidobacterium* and *Bacteroides* being relatively delayed. This early ecological imbalance of the gut microbiome affects the developing gut, brain, and immune system, and may lead to necrotizing enterocolitis (NEC), late-onset sepsis, WMI, long-term atopic diseases, metabolic syndrome, neurodevelopmental disorders, and other conditions. Therefore, it is necessary to accelerate the colonization of the intestinal microflora of preterm infants and optimize the intestinal microflora of preterm infants to improve the outcomes of the nervous system and brain development.

## Mechanism of the MGBA

### Overview of the microbial-gut-brain axis

The microbial-gut-brain axis (MGBA) is composed of the CNS, autonomic nervous system, enteric nervous system, hypothalamic-pituitary-adrenal axis, vagus nerve, and intestinal microorganisms and includes participation from the immune, metabolic, and other systems (Deplancke and Gaskins, 2001). Within this axis, there is interaction between gut microbiota and the brain, as microbes produce neuroactive compounds to act on the brain. Additionally, the brain regulates the gastrointestinal tract and immune function through the brain-gut-microbiome axis (BGMA) and affects intestinal physiological function and microbial composition (Diaz Heijtz et al., 2011). There are several mechanisms behind the important role that intestinal flora plays in the MGBA. The first is the short-chain fatty acid (SCFA) pathway. Certain substances that enter the intestine are difficult to digest but can be degraded and fermented by intestinal microorganisms to produce a variety of fatty acids, including butyric propionic and other short-chain fatty acids that not only provide energy to the body but also affect the permeability of the blood-brain barrier, thus affecting the development and function of the nervous system. The second mechanism is the neurotransmitter pathway. Specific strains of intestinal flora can regulate the levels of certain central neurotransmitters (Hannan, 2016). As evidence of this, neurotransmitters such as 5-HT, norepinephrine, and dopamine found in the striata of the brains of GF mice are significantly different from those of normal mice (Diaz Heijtz et al., 2011), and the content of 5-HT in the blood of germ-free mice is approximately 60% lower than that of normal mice. However, 5-HT content can be considerable restored by improving intestinal flora in GF mice (Yano et al., 2015). An important inhibitory neurotransmitter in the CNS, GABA, is closely related to emotion and memory. In their study, Liang et al. (2017) used antibacterial drugs to induce intestinal flora dysfunction in young rats, showing the rats to develop chronic depression and memory loss in adulthood. Meanwhile, the expression of the hippocampal GABA-A receptor alpha 5 and delta subunits had decreased, suggesting that intestinal flora dysfunction may have adverse effects on emotion and cognition by affecting the expression of GABA receptors. Finally, cytokines and other mediators can send signals from the gut to the brain *via* the vagus nerve. In particular, it has been shown that after a meal rich in fats and carbohydrates, a special subgroup of intestinal cells named intestinal endocrine cells releases hormones and peptides such as serotonin, cholecystitis, glucagon-like peptide-1, and others (Williams et al., 2014). These peptides bind to odontoid receptors located in the nucleus tractus solitaris and the hypothalamus to produce satiety and regulate energy expenditure (Latorre et al., 2016). The hypothalamic-pituitary-adrenal axis is related to physiology, psychology, and stress, and is the most important component of the body’s neuroendocrine system. Intestinal flora can influence behavioral responses *via* this axis. Thus, colonization by intestinal microorganisms is crucial for the development of the hypothalamic-pituitary-adrenal axis and can effectively stimulate the establishment and improvement of the central hypothalamic-pituitary-adrenal axis during gradual colonization and stabilization of microorganisms (Dinan et al., 2015). Taken together, the gut microbiome and brain are strongly interconnected, communicate in a variety of ways, and work together to maintain homeostasis.

### Gut microbes in the relation to the blood-brain barrier and nerve cells

#### Relationship between gut microbes and the blood-brain barrier

The formation of the blood-brain barrier plays a crucial role in the development of the nervous system (Dahl et al., 2018). Tight junctions are an important basis for maintaining the structure and function of the blood-brain barrier, and tight-junction protein-5 is a key target for regulating the permeability of cerebrovascular endothelial cells (Ochoa-Repáraz and Kasper, 2016). Studies have shown that the increased permeability of the blood-brain barrier in GF mice in early post-neonatal and adult life is primarily related to the low expression of endothelial tight junction proteins, including occludin and claudin-5 junction proteins, and that the integrity of the blood-brain barrier in GF mice can be effectively improved by supplementing Clostridium butyrate or butyrate (Ochoa-Repáraz and Kasper, 2016). Postnatal colonization of microbiota can also improve the permeability of the blood-brain barrier (Braniste et al., 2014), confirming that the microbiota plays a key role in the development of the blood-brain barrier.

#### Relationship between gut microbes and nerve cells

There is evidence that gut microbiota influences neurogenesis, as this process differs between GF and specific-pathogen-free (SPF) mice. Compared to SPF mice, brain-derived neurotrophic factors (BDNF) controlling neuronal survival are reduced in GF mice (Ichim et al., 2012), demonstrating that gut microbes can influence neuronal differentiation and ultimately brain development and health. Differing from neurons, microglia cells are responsible for the immune defense of the CNS and play a key role in brain development, participating in OL maturation and synaptic development (Erny et al., 2015). The interaction between intestinal brain axis and astrocytes and OLs may be mediated by microglia. In the model of chronic disease induced by oral antibiotics, intestinal microbiota modifies oligodendrocyte gene expression by affecting the synthesis of microglial metabolites, thus causing neurobehavioral abnormalities (Matcovitch-Natan et al., 2016). SCFAs may not only act directly on the microglia themselves but also indirectly affect the inflammatory environment in the brain through peripheral lymphocytes (Yan and Ajuwon, 2017). The activation of microglia is accompanied by an increase in cytokines and the metastasis of inflammatory cells, which promotes the release of tumor necrosis factor-α, interleukin (IL)-6, and other inflammatory factors that have a direct toxic effect on OLs, or directly mediates the destruction of OLs by other substances. Using a sterile mouse model, Lu et al. (2018) found that the effect of the microbiome on the development of early neurons and OLs may be mediated by its effects on neuroinflammation and insulin-like growth factors. Another study showed that the metabolites of gut microbes regulate astrocyte activity through the aromatic hydrocarbon receptor pathway, thereby affecting the inflammatory response of the CNS (Rothhammer et al., 2016). Regarding the gut-brain axis, metabolites released by the gut microbiome also play an important role in myelination in preterm infants. Chen et al. (2019) reported that antibiotic use can lead to demyelination, and others showed that transplantation of the microbiome can reverse the changes in these myelin-activating genes (Hoban et al., 2016).

## MGBA and neurological diseases

### MGBA and neurological diseases in adulthood

New research suggests that the malfunctioning of gut microbiota contributes to neurodevelopmental and neurologic disorders in humans and rodents including autism, ischemic brain disease, and Alzheimer’s disease (Luo et al., 2018). For example, the stools of patients with Alzheimer’s disease was analyzed by 16sRNA sequencing technology, and it was found that the diversity of intestinal microbes had significantly changed (Clarke et al., 2014). Additionally, *Bacteroidetes* in the feces of these patients had increased notably, and the increased *Bacteroidetes* were able to increase systemic bacterial lipopolysaccharide, promote inflammation and degeneration of the CNS, and reduce the content of SCFAs so as to increase the formation and aggregation of amyloid beta in the brain (Clarke et al., 2014). Compared with healthy adults, significant differences in the composition and content of intestinal flora in children with depression have also been shown, and the relative abundance of *Firmicutes*, *Actinomyces*, and *Bacteroidetes* was significantly changed (Zheng et al., 2016). GF mice transplanted with intestinal feces from depressed patients showed depressed-like behavior (Zheng et al., 2016), and analysis of fecal metabolites in depressed women showed that butyric acid content was significantly lower than that in healthy women (Thomas et al., 2012). Additionally, as the pathological feature of Parkinson’s disease (PD) is the aggregation of alpha synaptic nucleoproteins in the brain (Perez-Pardo et al., 2017), Kim et al. (2019) have demonstrated that alpha synaptophysin was transmitted from the gut to the brain *via* the vagus nerve. In PD model mice, probiotics can be used to restore the ecological imbalance of intestinal microorganisms, and the gut-brain axis can act on the brain to reduce anxiety and depressive behaviors and improve cognitive function (Caputi and Giron, 2018; Sun and Shen, 2018). Moreover, fecal microbial transplantation improved intestinal microbial dysregulation and had a neuroprotective effect on PD-model mice by inhibiting Toll-like receptor 4/TNF-α signaling pathway activation in the gut and brain (Sun et al., 2018).

### MGBA and neurological diseases in childhood

Desbonnet et al. (2014) have observed autism spectrum disorder (ASD)-like behavior in GF mice through a three-box social experiment, and their social behavior can be significantly improved by feeding them with probiotics. In addition, De Angelis et al. (2015) analyzed the fecal flora of children with autism spectrum disorders and normal children and found that the number of *Bifidobacterium* decreased in the intestinal microbes of children with ASD, while that of *Clostridium* increased.

### MGBA and neurological diseases in the neonatal period

Ni et al. (2019) reported that hypoxic-ischemic encephalopathy model rats treated with pilose antler polypeptide for 3 weeks demonstrated reduced brain damage, tissue inflammation, and OS. In an analysis of the changes of intestinal flora and its metabolites and SCFAs by qPCR and 16sRNA sequencing, these effects were found to be related to the improvement of intestinal barrier function by intestinal flora and the increase of strains producing SCFAs (Ni et al., 2019). The regulation of tight junctions of brain endothelial cells by the intestinal microbiome may influence the development of intraventricular hemorrhage. Additionally, intestinal microecological imbalance is an important risk factor for NEC and may lead to WMI. In the course of NEC, the inflammatory response caused by intestinal ischemia-reperfusion injury can cause hypoxia and ischemia in the brain, affect the formation of brain axons and myelin such as in the brain stem and central auditory pathways, and lead to neurophysiological disorders (Jiang et al., 2010). Recent research on the pathogenesis of WMI in premature infants has shown intestinal microorganisms to play an important role in its pathogenesis. The possible mechanisms behind this effect are elaborated upon in the following content.

## Potential mechanism of the MGBA in preterm infants with WMI

The most serious consequence of WMI in premature infants is periventricular leukomalacia which manifests as a large area of cystic necrosis adjacent to the ventricular wall. The main pathological types of WMI in preterm infants are as follows: focal cystic necrosis, focal microscopic necrosis, and diffuse non-necrotizing lesions. These three pathological types are associated with various clinical factors that affect the hemodynamic stability and inflammatory status of newborns before or after birth such as prenatal infection (maternal chorioamnionitis), postnatal infection, hypoxic ischemia, hypocapnia, hypoxemia, metabolic acidosis, and hypoglycemia (Back and Miller, 2014). Immature cerebral vascular anatomical structure, automatic regulation disorder, and exposure to exogenous injuries (such as hypoxia, ischemia, and inflammation) make immature brains especially vulnerable to injury (Schneider and Miller, 2019). With the improvement in perinatal care, the common form of WMI has changed from cystic to diffuse WMI (Back, 2015). The white matter area of premature infants is mainly composed of OLs, and OL in the white matter can be divided into four different continuous development stages: early OL precursor, late OL precursor, immature OL, and mature OL. Immature pre-OLs migrating from OL precursor cells eventually transform into mature OLs, and eventually generate the myelin sheath that covers axons. Pre-OLs in preterm infants are the main cellular targets in the brain, which are sensitive to hypoxia and inflammation and most vulnerable to damage (van Tilborg et al., 2018). If these newly produced pre-OLs do not differentiate into mature OLs (Segovia et al., 2008), then WMI and ultimately myelin dysplasia occur. Normal myelination is essential for axonal conductivity and plays an important role in the development of the cerebral cortex and later in learning and memory (Wang et al., 2020). The arrest of OL precursor maturation in WMI is accompanied by diffuse microglial activation. Microglia, the macrophages of brain tissue, represent the most abundant resident innate immune cells in the CNS. Microglial activation can be divided into two main forms: M1 (pro-inflammatory) and M2 (anti-inflammatory), both of which play an important role in the development of WMI and are related to its severity. After activation, M1 microglia phagocytose proliferates and migrates to the injured area like macrophages (Bokobza et al., 2019). At the same time, these cells can produce free radicals and pro-inflammatory cytokines, which aggravate neuroinflammatory damage and cause damage to the OLs and neurons. Transformation of M2 microglia into the pro-inflammatory M1 phenotype may lead to disruption of OL maturation.

It is also worth noting that intestinal flora colonization is concurrent with neural development (Borre et al., 2014; Sharon et al., 2016). Preterm birth leads to delayed intestinal bacterial colonization and dysbiosis, thus disrupting the normal relationship between intestinal flora and brain development during critical periods, leading to early brain damage and abnormal neuro programming, as well as unfavorable long-term neurodevelopmental outcomes. WMI in premature infants is the main cause of long-term complications in many chronic neurological diseases, which may lead to conditions such as cerebral palsy, mental retardation, audio-visual impairment, or motor retardation, increasing the burden placed on family and society. There is no specific clinical manifestation of WMI in premature infants; therefore, its prevention and early intervention have arisen as novel clinical problems. In the MGBA, intestinal microorganisms may reduce WMI in preterm infants through a variety of potential pathways, so as to promote the functional recovery of neural brain cells in preterm infants.

### Effects of metabolites on the MGBA in WMI

The changes in microbial metabolites reflect the changes in the microbiome, environment, and host (Krautkramer et al., 2021). The differentiation of pre-OLs and myelination is important in WMI, and the gut microbiota can influence myelination in different ways. In the gut-brain axis, microbiota-released metabolites also play an important role in myelination in preterm infants. SCFAs are the main products of fermented dietary fiber, including acetic, butyric, and propionic acids, and can improve the dysfunction of microglia in GF mice (Erny et al., 2015), suggesting that microbial metabolites may influence the communication of the gut and brain. Studies have shown that SCFAs derived from the microbiota can affect immune and inflammatory regulation (Corrêa-Oliveira et al., 2016). SCFAs can modulate innate immune cells such as neutrophils, dendritic cells, and microglia, as well as the adaptive immune system, subsequently affecting inflammatory processes (Rodrigues et al., 2016). By promoting the production of cytokines such as the tumor necrosis factor (TNF)-α, SCFAs directly regulate neutrophil differentiation (Rodrigues et al., 2016). SCFAs can inhibit the expression of key transcription factors, thus preventing the maturation of macrophages, monocytes, and dendritic cells and impinging on their ability to produce inflammatory cells or cytokines (Dalile et al., 2019). A known potential mechanism of SCFAs in WMI is the ability to inhibit histone deacetylase (HDAC) (Waldecker et al., 2008; Soliman and Rosenberger, 2011). Histone acetylation can regulate the expression of many genes and activate the transcription process. In contrast, HDAC leads to transcriptional silencing of chromatin. SCFAs are natural HDAC inhibitors, and HDAC-induced chromatin transcriptional silencing is reversed in the presence of SCFAs (Fang et al., 2021). It has been reported that sodium butyrate treatment can induce histone acetylation and further contribute to OL differentiation and maturation, thus exhibiting a protective effect on the brain in neonatal hypoxic-ischemic WMI rat models (Huang et al., 2018). Additionally, in neonatal hypoxic-ischemic WMI rat models, sodium butyrate attenuated brain injury by inhibiting IL-1β and chemokine CXCL10 production (Jaworska et al., 2017). Sodium butyrate has also been reported to transfer microglia in CNS injury models to the inflammatory suppressive phenotype M2 through HDAC effects (Tanoue et al., 2016; Jaworska et al., 2019). SCFAs can also regulate the adaptive immune system; for example, they can affect the differentiation of regulatory T cells (Treg) (Veltkamp et al., 2006; Fang et al., 2021). Treg cells, marked by the expression of the FOXP3 transcription factor, play an important role in suppressing unwanted immune responses and maintaining homeostasis (Moes et al., 2010; Figliuolo da Paz et al., 2021). The differentiation, maintenance, and migration of Treg cells are regulated by various signals provided by the microbiota (Wang et al., 2018). Sodium butyrate activates microglia by activating protein kinase (Akt) and inhibiting histone deacetylase (HDAC), inducing reversible prolongation of microglia processes. Prolongation of microglial protuberance has also confirmed the anti-inflammatory effect of sodium butyrate (Huang C. et al., 2018). Microbial metabolites may be involved in microglial pathophysiology as a biological signal and affect the immune responses of pro-inflammatory and anti-inflammatory microglial states. Crucially, SCFA production can stimulate microglial activity and alter the permeability of the blood-brain barrier. SCFAs affect the brain indirectly, mainly by activating the immune system and the peripheral nervous system.

### MGBA influences WMI through cytokines

Increasing evidence suggests that, in preterm infants, inflammatory cytokines are key mediators of WMI, and regulation of inflammatory responses may serve as a bridge between the microbiota and brain damage. A balanced relationship between the gut microbiome and the immune system is crucial for suppressing excessive inflammation. The immune systems and gut microbiota of premature infants are not fully developed, which can lead to excessive inflammation and negative effects on the brain. Cytokine attack can lead to programmed death of OLs in the periventricular white matter. Reports suggest that the inflammatory response induced by endotoxemia promotes the release of tumor necrosis factor-α, and the cytokines IL-6 and IL-β, resulting in vascular endothelial dysfunction and impaired cerebrovascular autonomic regulation, thus causing WMI (Lehnardt et al., 2002). WMI in preterm infants is caused by OL damage, which is in turn caused by microglial activation. The activation of microglia is accompanied by an increase in cytokines and a transfer of inflammatory cells, which promotes the release of more inflammatory factors, such as TNF-α, IL-1, and IL-6, and has a direct toxic effect on OLs. This study found that probiotics can produce lysosomal enzymes, immunoglobulins, interleukins, and interferon and immune factors, causing T cells to shift to the Th1 pathway where they mediate cellular immunity and inflammation and inhibit the activity of the important intracellular signal transduction nuclear transcription factors such as NF-κB and MAP activating enzyme, thus reducing ring oxidase- 2 (Cox-2). Probiotics can also downregulate the release of TNF-α and IL-6, upregulate anti-inflammatory cytokines, and enhance the activity of antioxidant enzymes *in vivo* and *in vitro*. Additionally, they can inhibit Th cell differentiation, thus decreasing the expression of inflammatory factors, reducing the nerve cell edema caused by WMI, effectively protecting immunity, and promoting rapid recovery of WMI nerve function in premature infants (Diamond et al., 2011; Lee et al., 2017; Akhoundzadeh et al., 2018). Some studies have confirmed that *Lactobacillus* and other bacteria can increase the glutamate reuptake ability of adjacent OLs in the synaptic cleft by regulating cytokines, thus reducing excitotoxicity (Favrais et al., 2011). The treatment of mice with IL-1β resulted in an increase in the number of unmyelinated axons and of those damaged by WMI, and it has been speculated that IL-1β may inhibit the maturation of OLs by disrupting several transcription factors that may regulate the maturation of OLs (Kim et al., 2011). Furthermore, TNF has been reported to be a key mediator in promoting LPS-induced OL death (Seki et al., 2021). A recent study found that *Klebsiella* overgrowth is highly predictive of brain injury in preterm infants and is related to the pro-inflammatory immune environment of the CNS (O’Neill et al., 2013). Neonates with severe brain injury show polarization of pro-inflammatory T cells and expansion of γδT cells in peripheral blood, driven by the secretion of IL-17 and VEGF-α. IL-17A is an important signal for γδT cells to migrate to the CNS where they exacerbate brain damage (O’Neill et al., 2013). The gut microbiota and its metabolites have significant immunomodulatory potential, such as the ability to stimulate the increase in γδT cells that produce IL-17A. In addition to direct effects on pre-OLs, cytokines can cause WMI by increasing the permeability of the blood-brain barrier, damaging the vascular endothelium, and reducing cerebral blood flow. Microorganisms can be recognized by various Toll-like receptor host cells that can release a large number of cytokines such as IL-1 and TNF-α, which act on pre-OLs and cause disorder in their differentiation and maturation (Kim et al., 2011).

### MGBA influences WMI by alleviating oxidative stress

During the perinatal period, excess free radicals are released after inflammation, hypoxia, and ischemia-reperfusion, which may disrupt the normal REDOX state of cells and increase OS and its associated toxic effects. OL precursors lack the ability to deal with oxygen free radicals and are vulnerable to attack by oxygen free radicals, which mainly attack OL precursor mitochondria and activate apoptosis-inducing factors and caspase-3, thus leading to OL precursor apoptosis (Raju et al., 2017). Periventricular OS injury often occurs before myelination, which is when a large number of OL precursors appear (Blomgren and Hagberg, 2006). Pre-OL precursors are more susceptible to intrinsic and extrinsic factors of OS (Dietz et al., 2016). Pre-OLs can easily capture iron ions and be attacked by free radicals. After ischemia-reperfusion injury, iron ions are released into the extracellular space, resulting in damage to the periventricular white matter. The microbiota can adjust OS through the MGBA. Reactive oxygen species (ROS) are intermediates produced by chemical reactions involving oxygen molecules. OS is potentially neurotoxic, leading to oxidation of deoxyribonucleic acid, biomolecular damage, diffuse cell dysfunction, and eventually cell apoptosis. During the perinatal period, excess free radicals released after inflammation, hypoxia, and ischemia-reperfusion can disrupt the normal REDOX state of cells, thus increasing OS and its associated toxic effects. Genes that regulate OL maturation are activated by OS, and OS can impede OL differentiation by promoting global histone acetylation (Dewald et al., 2011; Spaas et al., 2021). OS can also cause the death of precursor OLs through the activation of the caspase system (Back et al., 2005). When WMI occurs, ROS increase abnormally. Additionally, after activation of pre-OLs, microglia, and astrocytes in the brain, oxygen consumption increases, and ROS generation requires various enzymes (Dumitrescu et al., 2018), generating many highly oxidative metabolites, which can be influenced by the gut microbiota. Furthermore, studies have shown that the increase in ROS levels and the enhancement of the inflammatory response may be regulated by certain special microbiota through the MGBA, and changes in the gut-specific microbiota may occur in brain injury caused by various etiologies (Bonaz et al., 2018). Studies have shown that butyrate, the main metabolite of intestinal butyrate bacilli, can further improve the function of nerve cells by reducing OS in the brain, attenuating inflammation, and reducing neuronal apoptosis which occurs mainly through phosphatidylinositol-3 kinases mediated by BDNF. PI3K/serine-threonine protein kinase (Akt), a central signaling pathway that regulates neuronal growth and metabolism (Niemarkt et al., 2019), has been widely reported to be involved in cerebral hypoxic-ischemic diseases. BDNF protects against ischemic brain injury by antagonizing excitatory amino acids, inhibiting the inflammatory response, and reducing apoptosis. As an inhibitor of HDAC, butyrate can improve spatial learning and memory ability in the body along with the activity of neural cell bodies may inhibit neural cell apoptosis in hypoxic-ischemic cerebral diseases and can exert neuroprotective effects (Wu et al., 2018). Both butyrate and propionate enhance the expression of tyrosine and tryptophan hydroxylases, which are involved in the synthesis of dopamine, norepinephrine, and serotonin. SCFAs also change chromatin structure, induce the production of glutathione, and increase COX-2 (Li et al., 2020). SCFA treatment of GF mice can reverse microglia maturation defects, and the mechanism of action may be related to the reduction of OS. With the production of a large number of free radicals, the content of malondialdehyde (MDA), a biomarker of OS, increases, leading to nerve cell apoptosis by damaging cell lipids and blocking the synthesis and transport of genetic material. Probiotics can reduce MDA content and restore it to baseline levels. Studies have also found that pretreatment with tyrosine bacilli can inhibit apoptosis of antioxidant enzyme activity and reduce brain damage in mice (Sun et al., 2016). After supplementation with probiotics, peroxide dismutase, glutathione peroxidase, manganous-containing pseudocatalase, thioredoxin reductase, nicotinamide adenine, and dinucleotide oxidase can be produced to remove activated oxygen free radicals and reduce brain damage (Talwalkar et al., 2003; Bruno-Bárcena et al., 2004; Serrano et al., 2007). Therefore, the interaction between the gut and the CNS is bidirectional, and intestinal microbiota disorder is speculated to be the cause and effect of the enhanced OS response in the CNS.

**FIGURE 1:**
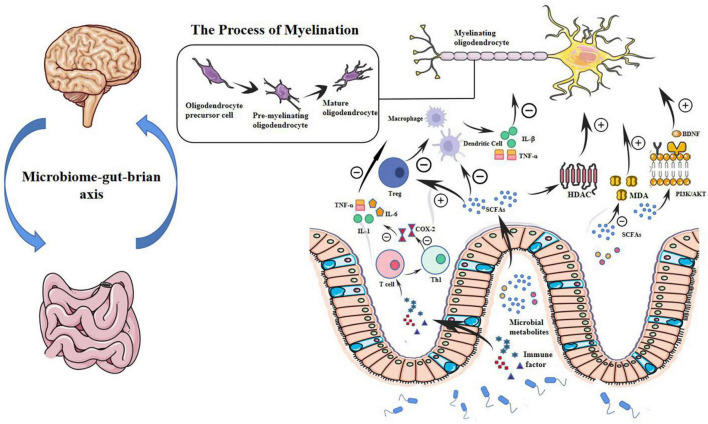
Microbial metabolites may modulate WMI in preterm infants through a potential microbial-gut-brain axis pathway. Myelination is determined by the normal maturation of pre-OLs and plays a key role in WMI. In the gut-brain axis, microbial metabolites such as SCFAs that inhibit HDAC can regulate OL maturation and subsequent myelination. SCFAs also regulate immune cells such as macrophages, T cells, and dendritic cells. SCFAs impair the production of cytokines, such as interleukin-1β and TNF-α. By modulating immune cells and cytokines, microbial metabolites can further regulate OL maturation and protect very preterm infants from WMI. Microbial metabolites also play a role in reducing oxidative stress in the brain through a BDNF-mediated PI3K/AKT signaling pathway. Probiotics can reduce MDA content and restore it to baseline, reducing oxidative damage. Probiotics can also produce immune factors, transfer T cells to the Th1 pathway that mediates cellular immunity and inflammation, reduce the production of COX-2, and downregulate the release of TNF-α, IL-6, IL-1β, and IL-1. pre-OLs, pre-myelinated oligodendrocytes; OL, oligodendrocyte; WMI, white matter injury; HDAC, histone deacetylase; TNF-α, tumor necrosis factor -α; BDNF, brain-derived neurotrophic factor; PI3K/AKT, phosphatidylinositol 3-kinases/serine-threonine protein kinase; MDA, malondialdehyde; IL, interleukin; SCFA, short-chain fatty acid.

This evidence suggests that the microbiota and its metabolites may regulate inflammatory and OS processes in the preterm brain, as shown in Figure 1.

## Potential microbiological treatments for WMI in preterm infants

### Probiotic therapy

As living microorganisms, probiotics can provide health benefits to the host (Martin and Walker, 2008). Probiotics not only improve the digestive tract, promote antitumor activity, delay aging, and relieve anxiety (Kane and Kinzel, 2018), but also exert important protection and repair effects on WMI nerve cells in preterm infants (Keunen et al., 2015). *Bifidobacterium longum, Lexobacillus swistifolia, Bacillus rhamnosus, Lexobacillus plantarum*, and *Lexobacillus casei* have been shown to effectively improve anxiety/depression and other neurological conditions in animal model species. After the appearance of WMI in preterm infants, *Lactobacillus*, *Bifidobacterium*, and *Fusobacterium* were significantly lower, while *Escherichia coli* in the feces increased significantly due to an impaired brainstem, hypothalamic-gastrointestinal neuromodulatory reflex, and imbalance of intestinal flora. Gastric emptying and intestinal peristalsis were further decreased, abdominal distension and dyspepsia were aggravated, and the absorption capacity of nutrients was weakened (Vizzini and Aranda-Michel, 2011; Yu et al., 2011). Treatment with probiotics can promote the colonization of normal intestinal flora in premature infants with WMI, increase the secretion of gastrin and motilin, increase gastrointestinal motility, help avoid abdominal distension, vomiting, and gastric retention, and improve feeding tolerance and milk production. Probiotics reduce WMI in preterm infants as they alter gut microbes and regulate the immune system and the inflammatory response. Probiotics can also secrete a variety of digestive enzymes such as hydrolyzing proteins, sugars, and fats to promote the digestion and absorption of food, ensure the metabolic balance of sugars and proteins in the body, and increase the absorption of nutrients needed to recover nervous function. Additionally, acidic substances such as lactic acid are produced by their metabolism and can reduce the pH value of the intestinal tract and inhibit the growth of pathogenic bacteria. Probiotics such as *Lactobacillus* can increase mucin secretion, improve barrier function, and compete with pathogenic bacteria and prevent their adhesion, thus inhibiting the production of toxins by pathogenic bacteria. With the correction of intestinal microbial imbalance, the gastrointestinal environment and function are improved nutritional intake is balanced and sufficient, nutritional status and immunity of the body are improved, the incidence of metabolic disorders and nosocomial infections in children is reduced, and the recovery of neural cell function and the outcome of WMI disease in preterm infants are promoted (Cheled-Shoval et al., 2014). Pregnant mice treated with *Lactobacillus acidophilus* and *Bifidobacterium infantile* have shown development of OL progenitor cells, inhibition of IL-1β-induced systemic inflammation, and attenuation of microglial activation in their offspring (Lu et al., 2020). The prophylactic use of probiotics in preterm infants can also reduce the incidence of NEC (Bin-Nun et al., 2005; Lin et al., 2008; AlFaleh and Anabrees, 2014; Beghetti et al., 2021). Since neurodysplasia is associated with NEC pathogenesis, probiotics have been hypothesized to reduce neurological injury (Adams-Chapman, 2018; Niño et al., 2018; Davani-Davari et al., 2019).

### Prebiotic therapy

Prebiotics are microbial nutrients that regulate the intestinal microbiota by promoting the growth of beneficial bacteria such as *Bifidobacterium* (Eiwegger et al., 2010). Prebiotics regulate immune cells, including T cells, neutrophils, and dendritic cells, to maintain immune balance and modulate inflammatory responses, which may later affect myelination and WMI in preterm infants (Eiwegger et al., 2010; Jeurink et al., 2013). Therefore, prebiotics are believed to reduce the extent of WMI. However, of two studies that investigated improved neurodevelopmental outcomes after prebiotic supplementation, neither found significant benefits (LeCouffe et al., 2014; van den Berg et al., 2016). Nonetheless, due to limited published data, it is difficult to determine whether probiotics or prebiotics can improve neurodevelopmental outcomes, and further studies are needed to elucidate their role in neurodevelopment.

### Treatment using fecal microecological transplantation

Fecal microecological transplantation has been an emerging biological therapy for the treatment of diseases related to intestinal microecology disorders in recent years and has been proven to be effective in the treatment of clinically refractory *Clostridium difficile* infectious diarrhea (Cammarota et al., 2017). However, the effect of fecal microecological transplantation on the treatment of nervous system disorders needs to be further explored.

## Conclusion

The parallel development windows and interaction between the gut microbiome and neurological system highlights the importance of the perinatal establishment of symbiotic gut microbes as a key step in optimizing brain development and long-term mental health. WMI is a multifactorial disease. Prenatal or postnatal harmful exposures, such as maternal infections, early nutritional intake, and medical interventions in the neonatal intensive care unit, can cause changes in the microbiota of preterm infants, all contributing to WMI. The gut microbiota may play a key role in the pathogenesis of this condition, and intervention in the microbiota of preterm infants may be a promising target for WMI treatment. In the future, the aims of neuroprotection and intervention may be achieved by regulating the intestinal microbiota of premature infants. In future studies, we hope to explore different pathways regarding WMI to build a more comprehensive explanation of the immune communication mechanism between the gut and the brain.

## Author contributions

LY, YuW, JZ, LZ, YiW, MZ, and CW prepared preliminary draft of the review. NZ and LY critically revised the manuscript for intellectual content. All authors contributed to the article and approved the submitted version.
